# OpenHSV: an open platform for laryngeal high-speed videoendoscopy

**DOI:** 10.1038/s41598-021-93149-0

**Published:** 2021-07-02

**Authors:** Andreas M. Kist, Stephan Dürr, Anne Schützenberger, Michael Döllinger

**Affiliations:** 1 Division of Phoniatrics and Pediatric Audiology, Department of Otorhinolaryngology, Head and Neck Surgery, University Hospital Erlangen, Friedrich-Alexander-University Erlangen-Nürnberg, Waldstr. 1, 91054 Erlangen, Germany; 2 Department of Artificial Intelligence in Biomedical Engineering, Friedrich-Alexander-University Erlangen-Nürnberg, Henkestr. 91, 91054 Erlangen, Germany

**Keywords:** Medical research, Oral anatomy, Diagnosis, Health services, Medical imaging

## Abstract

High-speed videoendoscopy is an important tool to study laryngeal dynamics, to quantify vocal fold oscillations, to diagnose voice impairments at laryngeal level and to monitor treatment progress. However, there is a significant lack of an open source, expandable research tool that features latest hardware and data analysis. In this work, we propose an open research platform termed OpenHSV that is based on state-of-the-art, commercially available equipment and features a fully automatic data analysis pipeline. A publicly available, user-friendly graphical user interface implemented in Python is used to interface the hardware. Video and audio data are recorded in synchrony and are subsequently fully automatically analyzed. Video segmentation of the glottal area is performed using efficient deep neural networks to derive glottal area waveform and glottal midline. Established quantitative, clinically relevant video and audio parameters were implemented and computed. In a preliminary clinical study, we recorded video and audio data from 28 healthy subjects. Analyzing these data in terms of image quality and derived quantitative parameters, we show the applicability, performance and usefulness of OpenHSV. Therefore, OpenHSV provides a valid, standardized access to high-speed videoendoscopy data acquisition and analysis for voice scientists, highlighting its use as a valuable research tool in understanding voice physiology. We envision that OpenHSV serves as basis for the next generation of clinical HSV systems.

## Introduction

**Figure 1:**
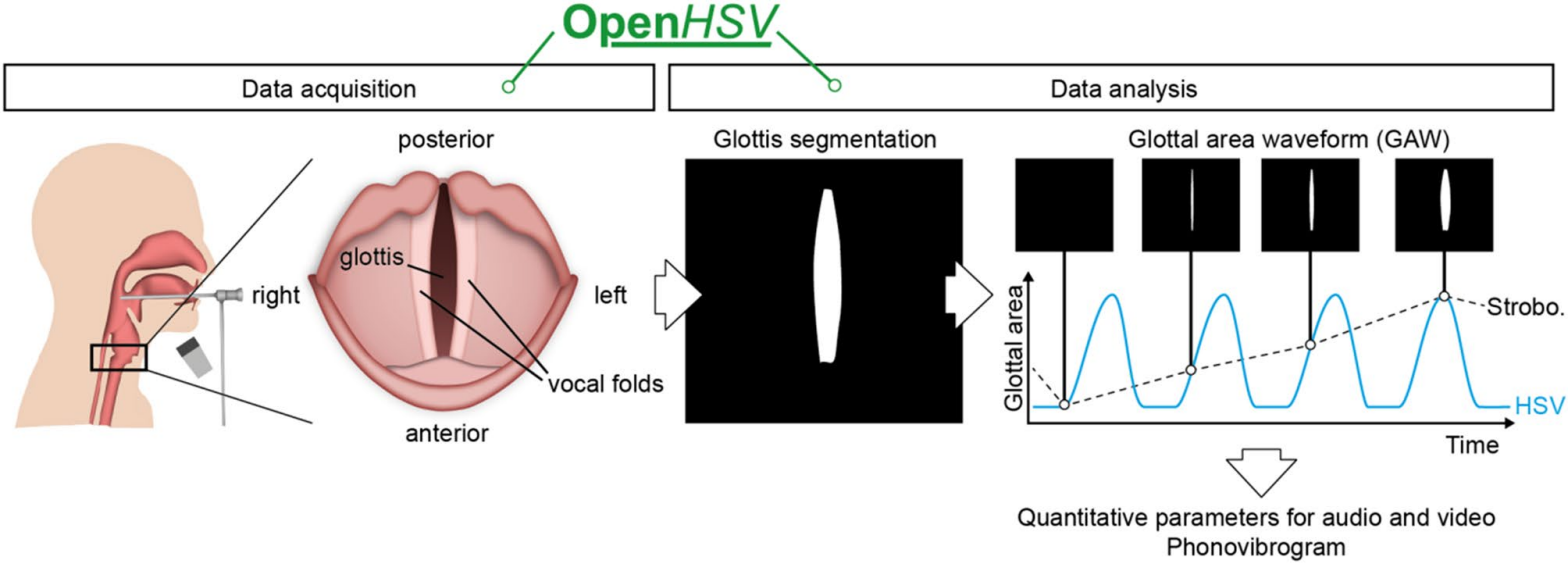
Laryngeal high-speed videoendoscopy is performed with a rigid endoscope yielding a top view of the larynx showing the vocal folds and the glottis. Glottis is segmented for each recorded video frame resulting in the glottal area waveform (GAW, blue). Stroboscopy is limited to single time points of individual cycles (dashed line). Using the GAW and the audio signal, quantitative parameters and the phonovibrogram are computed.

Laryngeal high-speed videoendoscopy (HSV) has been an emerging tool since decades in investigating voice physiology and pathophysiology^1^. The vocal folds, the main source of our voice and being located in the larynx (Fig. 1), are oscillating at very high frequencies. Typical fundamental frequencies for males and females are around 120 and 250 Hz, respectively^2^. According to the Nyquist–Shannon sampling theorem, the sampling rate has to be at least twice as high as the fundamental frequency to estimate the frequency. However, to observe the opening-closing transition within each cycle in an accurate and detailed way, a recent study suggests that sampling roughly 20-times higher, i.e. around 4000 Hz, is sufficient, given the average fundamental frequencies for humans^3^. Standard cameras are not able to acquire images at these high rates at full resolution. The current clinical gold standard uses a technique called stroboscopy. In stroboscopy, the fundamental frequency is computed from a high-resolution audio signal and the camera only acquires a single frame every n-th oscillation cycle (similar to shown glottal areas above the glottal area waveform (GAW), Fig. 1). This works well for healthy subjects with regular phonation, however, fails on irregular oscillations as often observed in patients^4–7^. In contrast, HSV acquires typically at 4000 fps or more^3,8^ and is therefore capable to resolve every oscillation cycle for low to very high phonation frequencies.

Despite the proven usefulness of HSV^9–11^, there have been only two commercially available HSV systems from KayPentax and Richard Wolf, that were launched years ago. Hence, in most cases HSV examinations are performed with either of the two, or very likely with unique research setups with custom hardware and custom software that is not standardized and often hinders comparability of results^4,12,13^. The main drawbacks of HSV, and we believe the reasons why HSV is still rarely applied in the clinic, are the high purchasing costs and the technical limitations, such as temporal and spatial resolution and sensitivity of the camera^1^, and, first and foremost, the needed complex analysis of the HSV footage^14–16^. In the analysis workflow, image processing, i.e. segmenting the glottis (Fig. 1), is a major bottleneck. Although fully automatic solutions for glottis segmentation have been proposed^17–20^, these methods have not seen further adaptation. With the advent of deep learning, however, this bottleneck has been successfully addressed^21–23^ and fast yet reliable solutions have been suggested^24^. Since several years, we have been developing a standalone analysis platform, Glottis Analysis Tools (GAT), that allows video and audio data analysis^15,16,25^. However, GAT is by design not interconnected with hardware and data acquisition. In summary, there is a lack of a unifying research platform that allows both, data acquisition and analysis, using state-of-the-art hardware and analysis tools.

In this study, we suggest a novel and open research tool that we term OpenHSV, that offers an examination-ready HSV hardware setup that acquires video and audio in synchrony and tested in a clinical environment. Additionally, we provide a user-friendly graphical user interface that implements a basic patient management system, an audio and video preview and acquisition feature, and a fully automatic data analysis platform based on state-of-the-art deep neural networks, providing a solid foundation for next generation clinical accredited, commercial systems^26^.

## Methods

### Hardware

**Figure 2:**
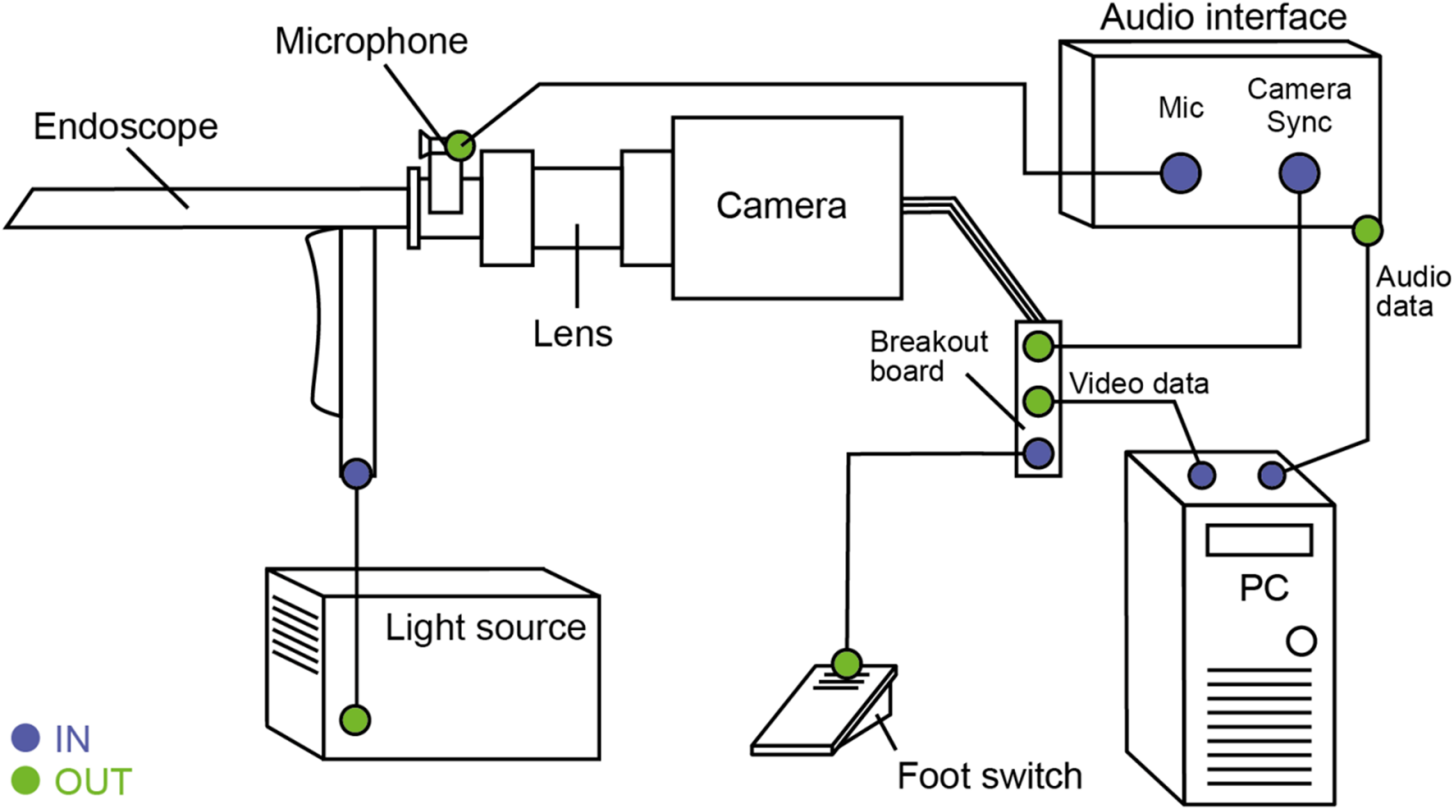
Connection scheme of the OpenHSV system. IN (blue) depicts entry of data, light or signal to a device and OUT (green) the exit of a data, light or signal from a device. The camera sends data to the computer and a reference signal to the audio interface to synchronize audio and video data. The audio signal is recorded via a high-quality microphone. A foot switch acts as an external trigger signal that stops the recording. The light source provides high power light via a light fiber to the endoscope. The endoscope is connected to a lens that relays the image to the high-speed camera.

The OpenHSV system is designed in a modular way to adapt to new hardware developments in terms of optics and technical equipment. In our study, a rigid, oral endoscope with 70° optics (Olympus), attached to a zoom lens (neomed) and connected to a color high-speed camera running at 4000 fps with a maximum ISO of 10,000 (IDT CCM-1540) is used. To determine a useful range of focal lengths, we tested different lenses from various suppliers (12 mm and 23 mm Karl Storz, 35 mm Richard Wolf, 80 mm Lighthouse, 15–25 mm neomed, see “Results”). Illumination is provided through a high power LED light source (Storz LED 300) connected via a light-fiber guide. Audio is recorded via a high-performance lavalier microphone (DPA 4060) connected to an audio interface (Focusrite Scarlet 2i2) using the XLR interface and is placed on a custom 3D printed microphone mount attached to the endoscope. The camera “Synch Out” signal is connected via a BNC to ¼ in TRS cable directly to one channel of the audio interface. The foot switch is connected to the “External Trigger In” port of the camera. An overview of the connection scheme of the individual parts is shown in Fig. 2. All components are connected to a standard commercial computer (Intel i5 processor, 16 GB RAM) equipped with an additional, current Gigabit ethernet card to connect the high-speed camera to the computer. We use deep neural networks that are optimized for CPU architectures and hence, no dedicated high-end graphics card is needed. However, when available, the graphics card is automatically utilized (see section “Data analysis”).

We further provide STL files online to 3D print custom holders for cables, the endoscope and the microphone. A droplet exposure protection shield, owing to the current COVID19 pandemic, to protect the camera is also available. These parts can be easily printed on a conventional stereolithography (SLA) or fused deposition modeling (FMD) 3D printers, where we found the latter faster and cheaper. We provide a tabular parts list in the supplement (Supplementary Table 1) and on the online documentation.

### Data acquisition

The examination, data acquisition and data analysis is performed using a dedicated graphical user interface (GUI) as described in a separate section. The high-speed camera is equipped with an on-board memory of 8 GB, allowing to record about 1.6 s at full spatial resolution and full speed (1440 × 1024 px and 4000 fps, respectively). During an examination, the video data is constantly written to a circular buffer on the on-board memory until an external trigger (e.g. a foot switch) is provided. By default, the trigger signal stops the recording, saving the last 1.6 s of footage. The camera provides a “Synch Out” signal that is an edge signal indicating the respective frame start. We refer to this signal as reference signal. We record the reference signal simultaneously with the audio signal to synchronize the video footage with the audio signal. Audio and reference signal are digitized at 80 kHz with 24 bit resolution.

After the external trigger, the acquisition of the video data stops immediately, the audio signal acquires another 1 s to ensure the correct alignment of video and audio. An acquired video can be previewed, the complete video or a fraction thereof selected, and downloaded from the camera to the computer. Video footage is saved in two ways, lossless and lossy for data analysis and portability, respectively. The data is stored as “.mp4” files using the h264 codec. Audio is saved as uncompressed “.wav” files. Patient, video, and audio metadata are saved as “.json” file. If data analysis was performed, the glottal area segmentations are saved as “.hdf5” files and quantitative parameters as “.csv” files.

### Audio and video signal alignment

**Figure 3:**
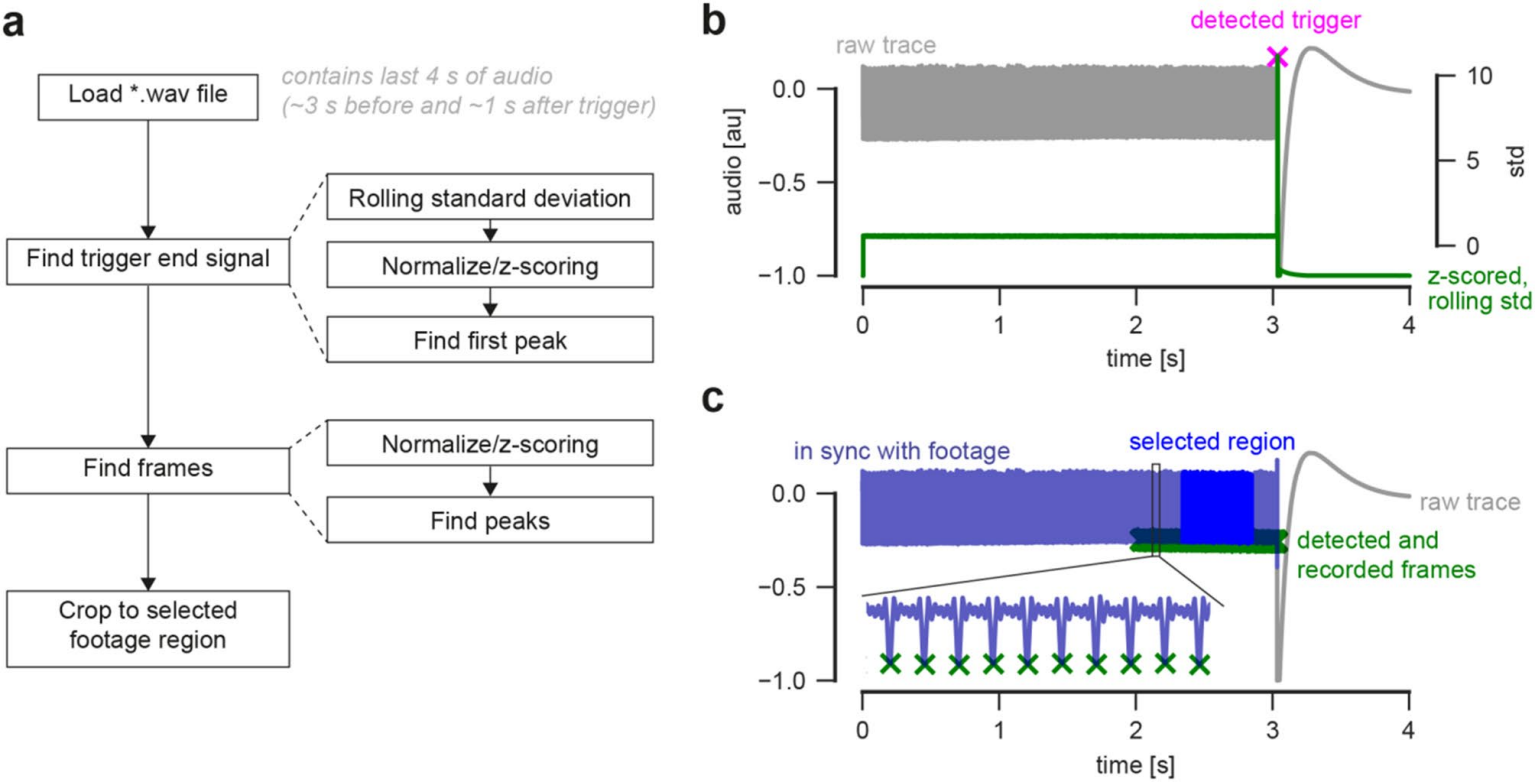
Audio–Video alignment. (**a**) Analysis pipeline. (**b**) Detection of end trigger using a normalized, rolling standard deviation (std) on example data. (**c**) Detection of recorded frames and extraction of selected area on the same example data as shown in panel (**b**).

The audio file contains the camera reference signal together with the subject audio signal. We use a multi-step analysis pipeline to align the audio signal to the camera frames (Fig. 3a). First, we compute a rolling standard deviation (std) using a 2.5 ms window of the raw reference signal. Next, we z-score the std signal and find the most prominent peak defining the end trigger event (Fig. 3b). Each frame is indicated by a peak in the reference signal (Fig. 3c). We detect the total recorded frames on the camera as peaks relative to the end trigger (Fig. 3c). The audio signal corresponding to the selected and transferred data is extracted and used for further analysis. We do not correct for the potential time delay between source generation and acoustic signal detection.

### Data analysis

Data analysis is performed individually for video and audio data (Supplementary Fig. 1).

#### Video

After acquisition and region of interest (ROI) selection, we implemented a fully automatic glottis segmentation based on established, efficient and CPU optimized deep neural networks^24^ trained on the openly available BAGLS dataset^22^. The BAGLS dataset contains 59,250 high-speed video frames with the corresponding glottis segmentation mask. The exact training process is described elsewhere^22,24^. Briefly, an optimized encoder-decoder network based on the U-Net architecture^27^ is trained to predict glottal area segmentations based on endoscopic images. After manually selecting an ROI around the glottis, the full data is downloaded from the camera and the ROI data is subsequently analyzed on a frame by frame basis by the deep neural network. The use of an ROI is recommended, as this accelerates significantly the network inference and removes variances of the distant image. We provide with OpenHSV a pre-trained network that is also individually accessible at our Github repository (https://github.com/anki-xyz/openhsv/cnn). The resulting glottal area waveform (GAW) is used as basis for further computations of quantitative parameters^28^ and is a one-dimensional function of all identified, i.e. segmented, pixels within one frame over time. We subsequently detect individual cycles in the GAW using standard peak finding algorithms as implemented in scipy^29^. For symmetry measures, we estimate the glottal midline at each maximum cycle using either image moments or principal component analysis in the segmentation mask similar to previous works^30^, also incorporating temporal context by summing adjacent frames to improve midline detection. We next identify the intersection of each glottal midline estimate with the segmented glottal area to find the anterior and posterior glottis points. Finally, we compute the phonovibrogram (PVG) as previously reported^31^ and the GAW for the left and right vocal fold as the area of left and right vocal fold to the estimated midline, respectively.

Video or image quality was assessed using the Natural Image Quality Evaluator (NIQE). The NIQE score is a blind, no-reference score that reports image quality based on the statistics of natural scenes^32^ and was already successfully applied to investigate laryngeal endoscopy image quality^33^. In general, the lower the NIQE score, the better the image quality. Briefly, the NIQE score is based on natural scene statistics extracted from undistorted images. These statistics were used to construct quality aware features that were themselves fitted to a multivariate Gaussian model serving as reference. The NIQE score then represents the distance between a multivariate Gaussian fit extracted from the test image and the aforementioned natural scene-derived multivariate Gaussian reference model. We computed the NIQE score using its implementation in scikit-video for the monochrome and the color images in the BAGLS dataset and for the OpenHSV-derived example images.

#### Audio

We similarly process audio signals to the GAW (see Supplementary Fig. 1a). First, we select the corresponding subset of the audio data in relation to the video data using the video reference signal acquired simultaneously with the audio signal (see audio and video signal alignment, Fig. 3). Next, we compute the fundamental frequency similar to the GAW (Table 1) to ensure validity of both signals (see also Supplementary Fig. 2).

**Table 1:** Clinical parameters contained in OpenHSV.

Clinical parameter	Source signal	References
Mean-Jitter	Audio, GAW	^34^
Jitter (%)	Audio, GAW	^35^
Mean-Shimmer	Audio, GAW	^34^
Shimmer (%)	Audio, GAW	^35^
Harmonics to noise ratio (HNR)	Audio	^36^
Cepstral peak prominence (CPP)	Audio	^37^
Open quotient (OQ)	GAW	^38^
Closing quotient (CQ)	GAW	^39^
Speed quotient (SQ)	GAW	^38^
Asymmetry quotient (AQ)	GAW	^40^
Rate quotient (RQ)	GAW	^38^
Speed index (SI)	GAW	^38^
Fundamental frequency (F0)	Audio, GAW	^38^
Amplitude perturbation factor (APF)	Audio, GAW	^41^
Amplitude perturbation quotient (APQ)	Audio, GAW	^41^
Glottis gap index (GGI)	GAW	^42,43^
Amplitude quotient	GAW	^44^
Stiffness	GAW	^45^
Amplitude symmetry index (ASI)	GAW	^46^
Phase asymmetry index (PAI)	GAW	^47^

### Quantitative parameter computation

Given the total GAW, the GAW for the left and the right vocal fold, and the audio signal, we compute quantitative parameters. In the initial release, we provide in total 18 clinically relevant parameters for the GAW and nine clinical parameters for the audio signal (Table 1). All parameters have been previously reported (see references in Table 1) and have been reported in detail for healthy subjects^48–50^. Individual detected cycles in video and audio data were used to compute jitter and shimmer measures, as well as all other GAW measures. The complete audio signal was used for harmonics-to-noise-ratio (HNR) and cepstral peak prominence (CPP). We used the partial GAW for left and right vocal fold to compute the amplitude symmetry index and the phase asymmetry index. A comprehensive overview of these parameters is given in Refs.^44,50,51^**.**

### Graphical user interface (GUI)

The OpenHSV GUI (Supplementary Movie 2) is written in Python 3.6 and mainly based on the libraries PyQt5 and pyqtgraph. The high-speed camera is interfaced using the camera manufacturer’s software developmental kit (IDT SDK). Video data are processed as multi-dimensional numpy arrays^52^. We interact with the audio interface via the sounddevice library. Patient data is recorded and saved to a local file system; the patient, video and audio recording metadata is further saved to a human-readable JSON file. The GUI provides a tabular overview of all recorded patients that further contains a search option to allow retrieving dynamically metadata from a given subset of patients. It gives also fast and easy access to previously recorded data, being for example important to visually compare multiple acquisitions at different time points of the same patient.

### Clinical study

We recruited 28 healthy individuals to perform a preliminary clinical study. All individuals were identified as normophonic, had no laryngoscopic organic or functional disorders and did not report any issues with their voice. All participants gave their written and informed consent. This study was approved by the local ethics committee at the University Hospital Erlangen (#290_15) and was conducted in accordance with respective guidelines and relevant regulations. All acquisitions were made with the same settings and equipment. We analyzed an at least 1000 frame long segment in each recording with at least 20 glottal cycles, as recommended previously^53^.

## Results

### Setup

**Figure 4:**
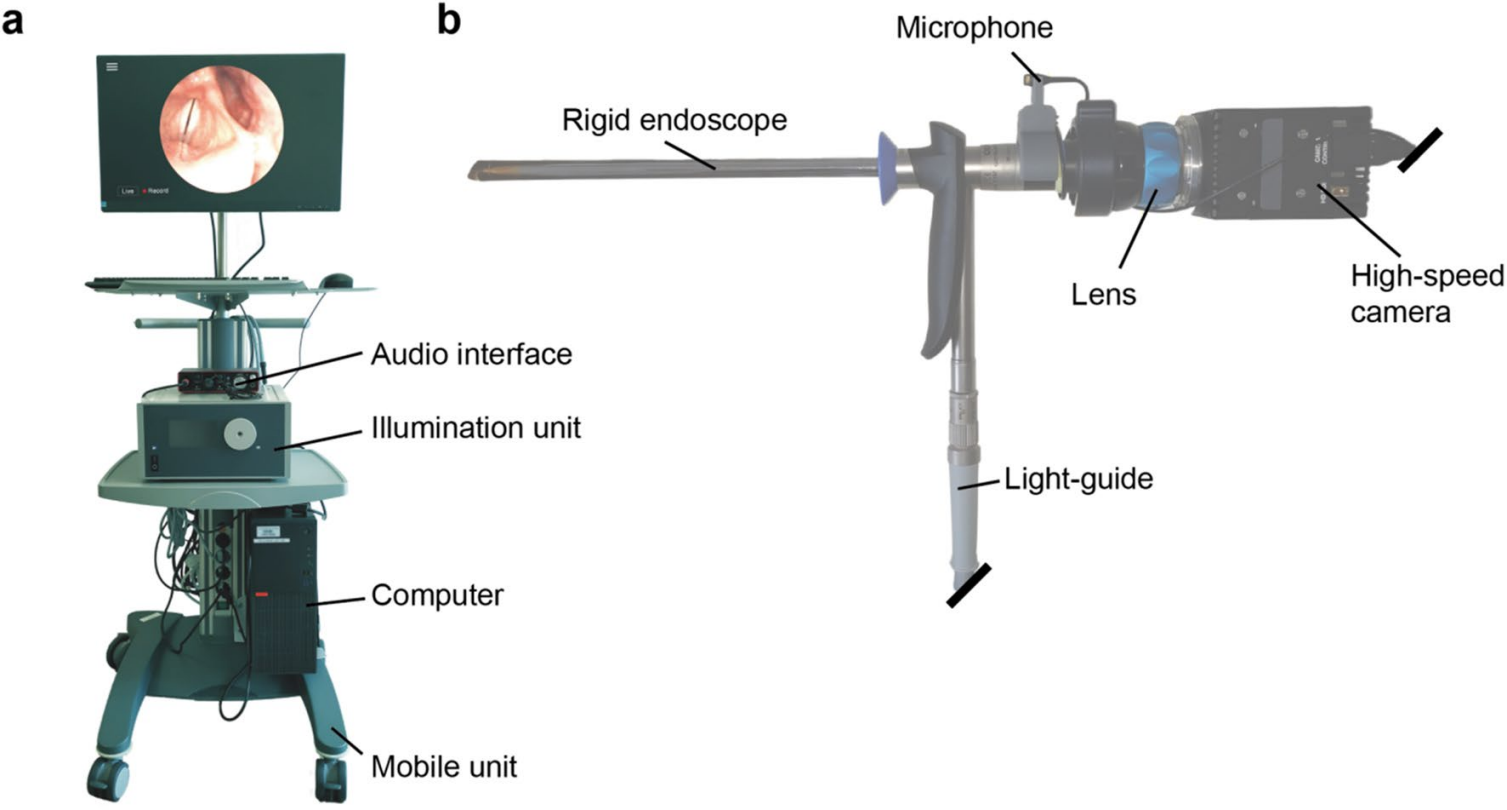
The mobile imaging unit. (**a**) The mobile equipment tower consisting of computer, illumination source, audio interface, monitor and human–device-interaction components, (**b**) the imaging unit consisting of rigid endoscope, microphone, lens, high-speed camera, and light-guide.

The OpenHSV setup consists of a mobile, equipment storage tower and a mobile imaging unit (Fig. 4). In particular, we use a mobile platform containing a typical consumer-grade computer to interact with equipment and to conduct examinations, an illumination unit for providing light and an audio interface to record audio and the camera synchronization signal (Fig. 4a). A consumer-grade, 23″ monitor together with keyboard and computer mouse that can be disinfected is used to interact with the software. The imaging unit as shown in Fig. 4b uses a rigid endoscope. The endoscope is connected to a lens and to the high-speed camera. The light-guide transmits light from the illumination unit to the endoscope to illuminate the larynx.

**Figure 5:**
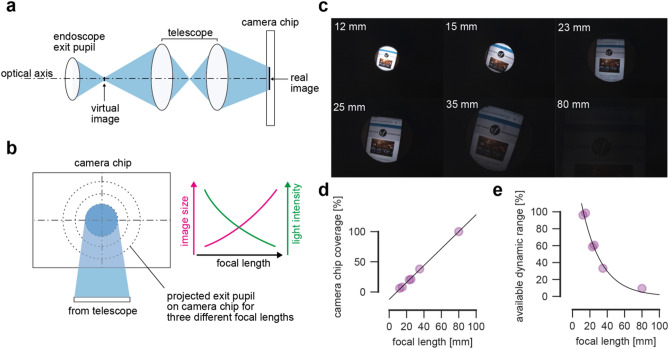
Image formation process. (**a**) Optical setup including the endoscope exit pupil, the lens simplified as telescope, and the camera chip. The real image is formed on the camera chip. (**b**) Image size on camera chip depending on the focal length. Focal length and image size vary proportionally; focal length and light intensity are inversely correlated. (**c**) Example images from the same scenery with lenses with varying focal lengths. (**d**) Chip coverage in percent vs. focal length. Black line indicates one-exponential fit. (**e**) Available dynamic range vs. focal length. Black line indicates one-exponential fit.

We first evaluated the image formation process from endoscope exit pupil to camera chip via a lens (basically an optical telescope) as these optics are crucial for a good image quality (Fig. 5a). Further, the lens’ focal length determines the image size, i.e. the pixels covered on the camera chip, and the signal-to-noise ratio, as a fixed amount of light is distributed across a varying surface (Fig. 5b). We found that a high-quality endoscope with 10 mm exit pupil diameter delivers large images together with a very high light intensity. We investigated different lenses with different focal lengths (12–80 mm) to determine the best trade-off between image magnification and signal to noise ratio. In Fig. 5c, we show example images from the same scene and the same recording settings with varying lenses and found, the larger the focal length of the lens, the larger the projected image size on the camera chip (Fig. 5c,d). In Fig. 5e, we show that the dynamic range of the images is higher the less the focal length is. In Supplementary Fig. 3 we show the dynamic range on example images and their respective intensity distribution histograms. Overall, low focal length lenses provide sharp images with satisfactory dynamic range. In case of the 80 mm focal length lens, there is no satisfactory image possible. However, we would like to point out that all measurements are due to the combination of endoscope, lens, camera and acquisition settings. In an examination scenario, we found that focal lengths up to 25 mm are a good trade-off between available dynamic range and image size.

### Clinical examination

**Figure 6:**
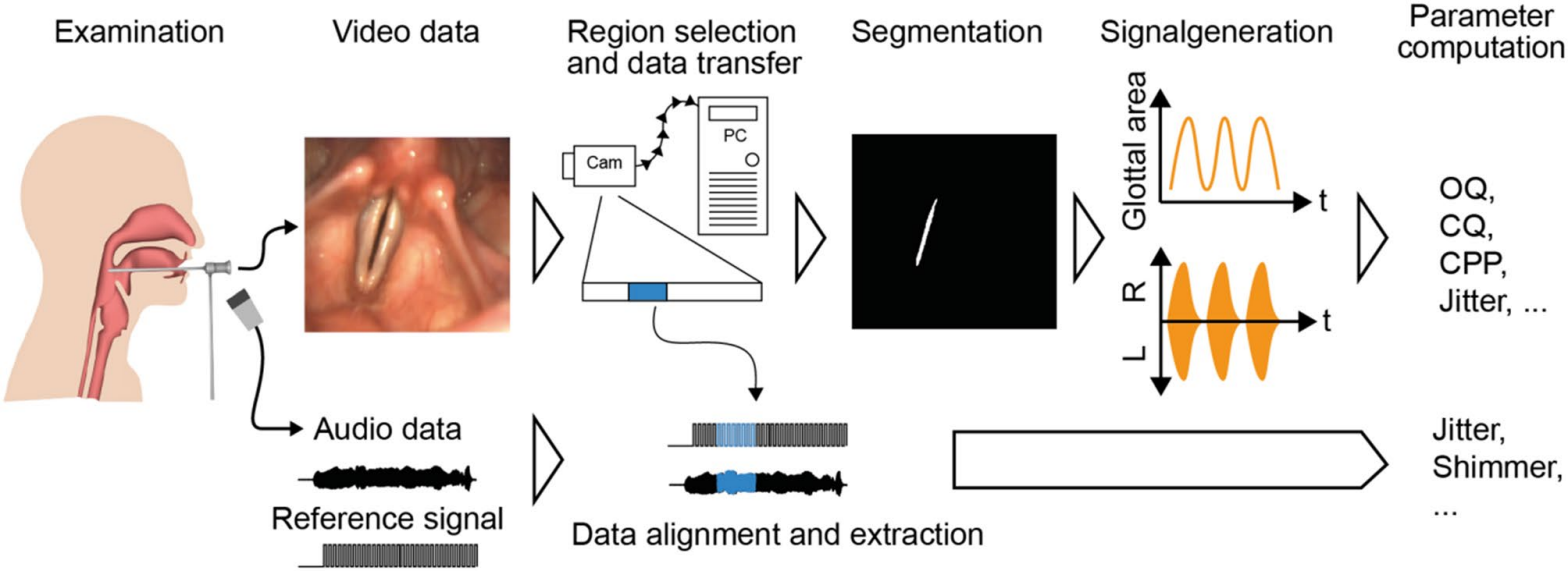
Examination and data analysis workflow. Audio and video data is acquired. Next, a subsection is selected and transferred to the computer. Using the video data, the glottal area is segmented and converted to signals that are used for parameter computation. The audio signal is aligned to the video footage using the reference signal and is subsequently analyzed.

**Figure 7:**
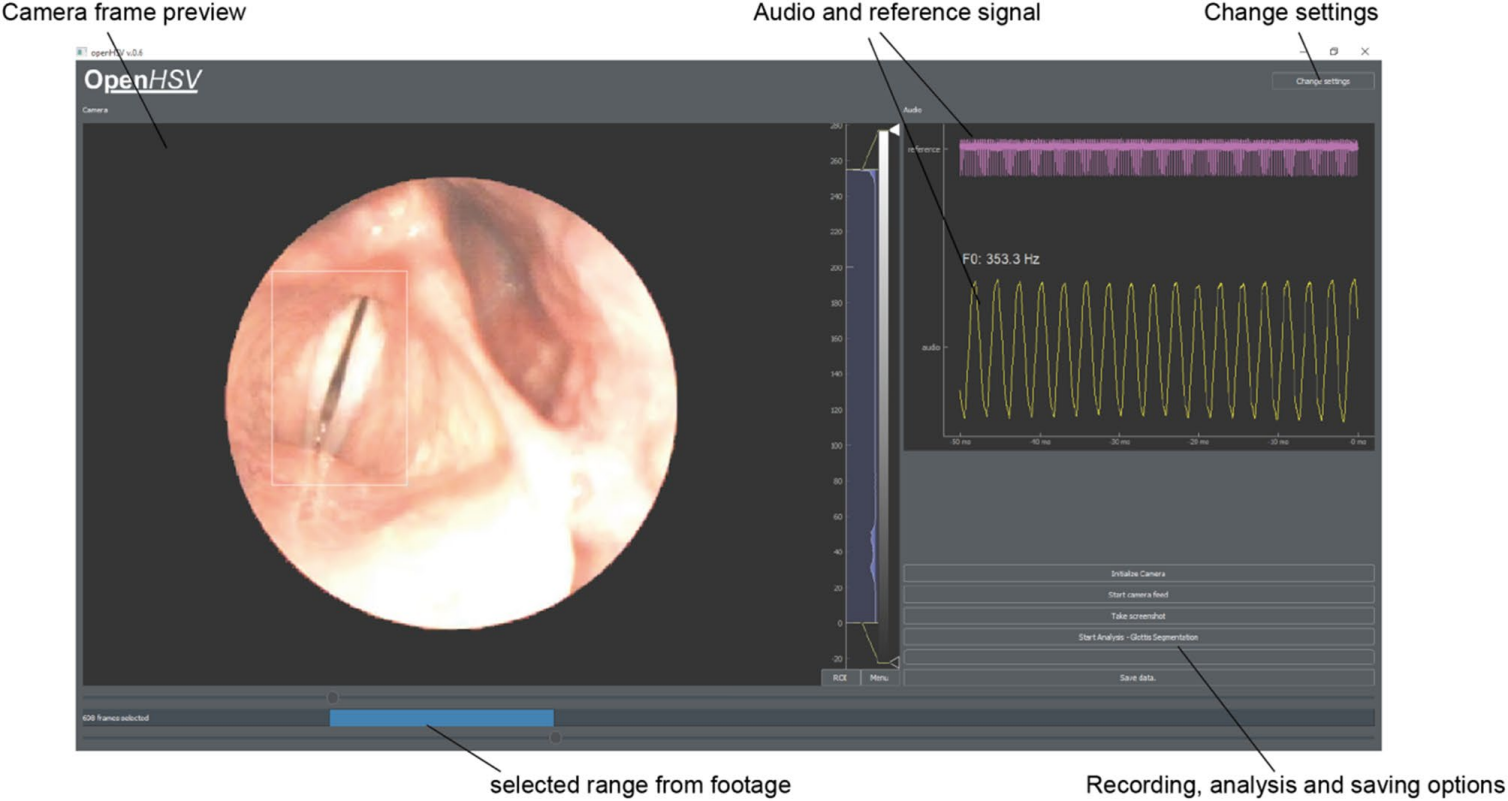
The OpenHSV graphical user interface. Camera image (left) and audio trace (right) are previewed online. The reference signal (pink) and the audio trace (yellow) are shown, together with the estimated fundamental frequency of the audio data. After an end-trigger (e.g. using a foot-switch), the user selects a footage range and is able to analyze and/or save the audio and video material and optionally analyzes the data directly.

We next tested the ability to record simultaneously video footage and audio data in a typical examination setting, and analyze the resulting data (Fig. 6). With the imaging unit shown in Fig. 4a, we performed examinations of healthy subjects. Using our custom GUI (Fig. 7), we are able to control the recording settings and receive a live feedback of the video and the audio data. As the footage can be pretty large (several gigabytes) and may contain not relevant information, such as sequences without phonation or swallowing artifacts, the examiner is able to select a subset from the whole recording. Selected video data will be transferred to the computer.

The video analysis is based on the segmentation of the glottal area. The segmentation is performed fully automatic using a deep neural network as described elsewhere^22,24^. The segmentation is further converted to the glottal area waveform (GAW). Next, we define the glottal symmetry axis fully automatically^30^ and convert the segmentation map into a phonovibrogram that allows a two-dimensional representation of the laryngeal dynamics^31^.

### Clinical validation

Our aim is to compare the OpenHSV recordings to data generated by established hardware and to validate our novel equipment and analysis platform. Therefore, we conducted a small-scale clinical study and analyzed 28 examinations from healthy individuals recorded with the OpenHSV system.

**Figure 8:**
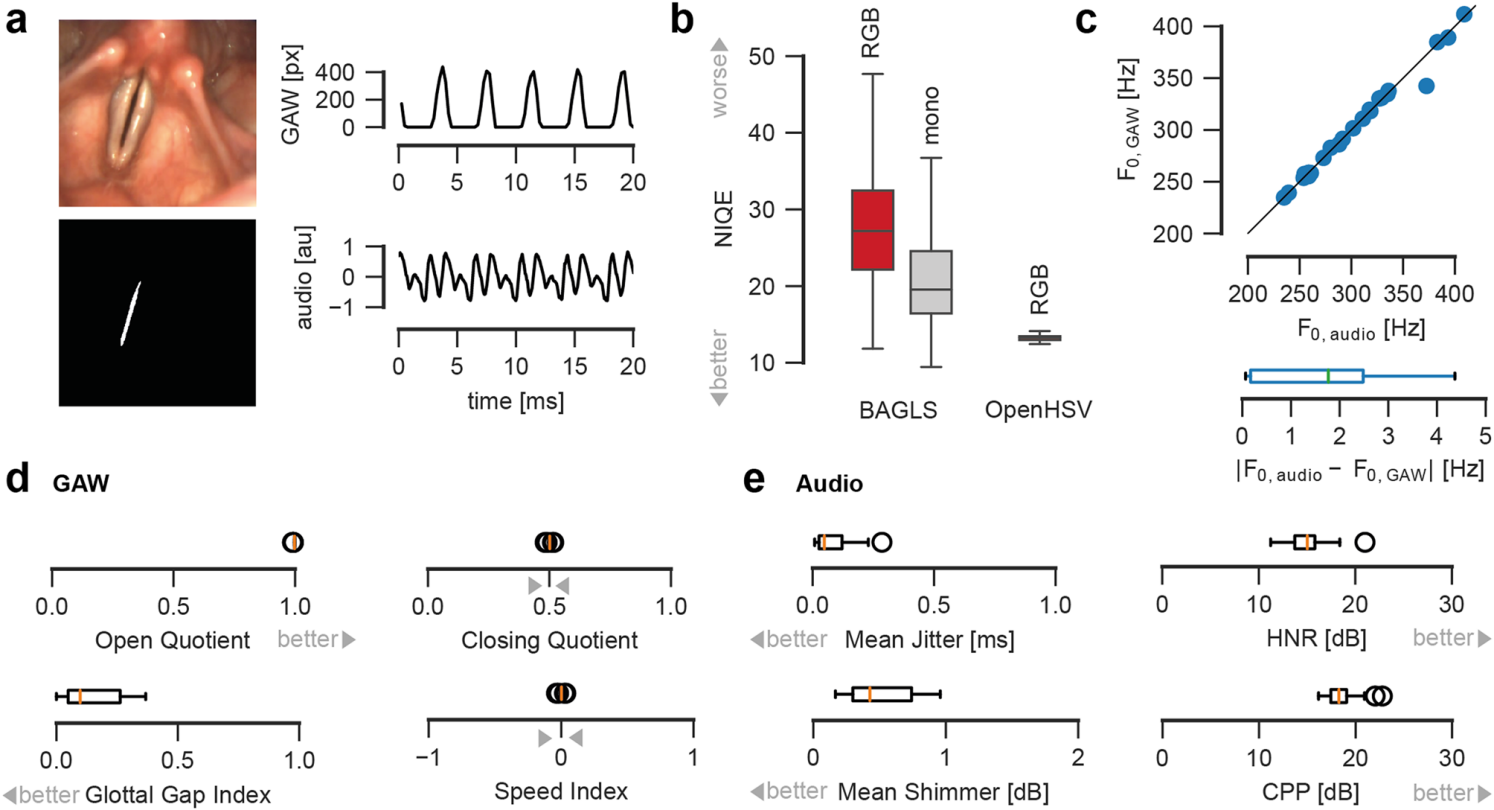
OpenHSV provides clinical relevant information. (**a**) Exemplary video, segmentation and audio data. (**b**) Image quality of OpenHSV compared to BAGLS dataset using NIQE. (**c**) Correlation of fundamental frequency determined in audio and video signal. Line indicates straight line of origin and perfect agreement between audio and GAW. (**d**) Exemplary GAW-derived quantitative parameters, namely Open Quotient (OQ), Closing Quotient (CQ), Speed Index (SI) and Glottal Gap Index (GGI). (**e**) Exemplary audio signal-derived quantitative parameters, namely mean Jitter, mean Shimmer, Harmonics-to-Noise-Ratio (HNR), and Cepstral Peak Prominence (CPP). Better values are indicated with gray arrow heads. For CQ and SI, 0.5 and 0 are desired values.

The subject age range was from 17 to 46 with a median age of 20. In Supplementary Fig. 4, we show representative images from the recordings. In Supplementary Movie 1, we show an example recording of 1000 consecutive frames as used in our analysis procedure. Using the analysis procedure depicted in Fig. 6 and described in the methods, we compute for each recording the raw endoscopy video, the corresponding segmentation maps, the glottal area waveform (GAW), and the corresponding audio and reference signal (Fig. 8a).

#### Image quality

We first determined the image quality of the OpenHSV system using the Natural Image Quality Evaluation (NIQE) score, a blind image quality metric that needs no reference images. We found that the OpenHSV system outperforms other imaging modalities that are contained in the BAGLS benchmark dataset that consists of a blend of data from seven different institutions having different equipment and recording conditions^22^. As shown in Fig. 8b, the mean NIQE for the OpenHSV System is 13.19 compared to the mean NIQE score of 28.79 and 22.42 for RGB and monochrome images in the BAGLS dataset, respectively. Even though that monochrome images pool color on each pixel and do not show interpolation artifacts due to the Bayer matrix, the image quality is still worse than the OpenHSV data (Fig. 8b).

#### Video-audio signal alignment

As the oscillating vocal folds are the main source of the phonation, the vocal fold fundamental oscillation frequency should be identical to the fundamental frequency determined from the corresponding audio signal. As shown in Fig. 8c, the fundamental frequencies are almost identical given the accuracy of our measurement systems showing typically deviations of less than 2 Hz (median 1.76 Hz) and are therefore negligible. Example audio and GAW power spectra of the analyzed recordings are shown in Supplementary Fig. 2.

#### Clinical quantitative parameters

We next computed clinically relevant parameters for healthy subjects that we implemented in OpenHSV. In general, the computed parameters (Tables 2, 3) have a similar magnitude as reported previously for healthy subjects^48–50^. We provide the distributions for a subset of GAW-derived and audio-derived parameters in Fig. 8d,e. In comparison to a recent study that focused on the analysis of HSV data of healthy individuals^50^, we found similar value distributions for parameters derived from the GAW, such as a similar open quotient (ours 0.998 vs. 0.927–0.999 reported) and asymmetry quotient (ours 0.501 vs. 0.511–0.554 reported). Similarly, the mean Jitter and mean Shimmer for the GAW signals observed (0.176 ms and 0.140 dB) are comparable with the aforementioned study (0.126–0.166 ms for mean Jitter and 0.102–0.130 dB for mean Shimmer^50^). We additionally observe on the audio data high values for HNR and CPP (on average 15.21 dB and 18.60 dB, respectively) which is an indication for healthy phonation (HNR on average 11.9 dB for normals^36^, CPP > 10 dB^54^). A good indication that both, video and audio, signals are in high synchrony are the similarities in fundamental frequencies between video and audio data (compare Tables 2 and 3, Fig. 8c). We therefore conclude that the whole system, consisting of experimental setup and analysis software, produces reliable and plausible results for the investigated healthy subjects.

**Table 2:** Glottal area waveform (GAW) parameters.

Parameter	Mean	std	Min	Max	Unit
Mean Jitter	0.176	0.070	0.072	0.375	ms
Jitter%	5.286	2.067	2.041	10.962	au
Mean Shimmer	0.140	0.127	0.035	0.440	dB
Shimmer%	0.257	0.249	0.059	0.889	au
Fundamental frequency (F0)	302	49	235	410	Hz
Open quotient	0.998	0.003	0.989	1.000	au
Closing quotient	0.504	0.044	0.402	0.567	au
Speed quotient	1.020	0.030	0.971	1.093	au
Asymmetry quotient	0.501	0.007	0.482	0.518	au
Rate quotient	1.027	0.033	0.973	1.129	au
Amplitude quotient	3.735	0.741	2.137	5.160	au
Speed index	0.003	0.014	− 0.035	0.035	au
Glottal gap index	0.150	0.126	0.000	0.367	au
Stiffness	0.278	0.065	0.186	0.431	au
Amplitude symmetry index	0.974	0.014	0.932	0.993	au
Phase asymmetry index	0.070	0.063	0.006	0.258	au

**Table 3:** Audio parameters. ^a^Amplitude perturbation quotient with varying windows sizes.

Parameter	Mean	std	Min	Max	Unit
Mean Jitter	0.079	0.072	0.009	0.286	ms
Jitter%	2.408	2.260	0.312	8.280	au
Mean Shimmer	0.590	0.603	0.167	3.095	dB
Shimmer%	2.093	1.964	0.623	10.283	au
Fundamental frequency (F0)	302	50	235	412	Hz
Harmonics-noise-ratio (HNR)	15.126	2.415	11.224	21.000	dB
Cepstral peak prominence (CPP)	18.596	1.629	16.160	22.771	dB
Amplitude perturbation factor (APF)	6.877	7.237	1.931	37.181	au
APQ3^a^	3.665	4.494	0.671	22.623	au
APQ5^a^	3.812	3.156	1.196	15.979	au
APQ11^a^	5.132	3.506	1.537	16.584	au

## Discussion

In this study, we suggest a new and open research hardware and software platform that we termed OpenHSV. OpenHSV’s software and analysis package is distributed open source and the hardware can be purchased commercially off-the-shelf. Using state-of-the-art components, we are able to acquire both, high quality audio signals and video footage. OpenHSV allows further the direct signal analysis and provides on time clinically relevant information. OpenHSV can be easily expanded by adding custom written Python code.

Medical equipment requires to be setup with low levels of expertise. Being a research tool, the setup of OpenHSV is non-trivial and needs attention. While we provide detailed instructions in our online documentation, personnel without basic knowledge in computer science (hardware and software installation) may have difficulties to setup OpenHSV. As we are happy to provide help, we highlight that OpenHSV is not a simple Plug&Play system. However, parts of OpenHSV, especially parts of the data analysis functionalities, have been integrated in commercial and clinical accredited systems, combining both, openness and easiness for future researchers and clinical examiners^26^.

High-speed videoendoscopy strongly relies on high-speed cameras. These cameras are highly specialized and various setup configurations are used^1,22^. Especially, cameras from the two existing commercial systems are very handy and have small camera chips. The size of the camera chip is indeed a limiting factor for image quality. The larger the individual pixel size, and the higher the desired resolution, the larger the camera chip (see also Fig. 5). As our endoscope exit pupil size and the amount of transmitted light is fixed, an image magnification worsens the signal-to-noise-ratio. Thus, cameras with a smaller sensor size are likely better suited. However, we were not able to find another camera that fulfills the inclusion criteria of acquiring at 4000 fps, state-of-the-art spatial resolution and low-weight body and small form factor, which are important features to be considered in camera selection.

A typical bottleneck of high-speed cameras is the data transfer from the camera to the computer. To allow high-resolution acquisitions, typically, high-speed cameras write the high-speed footage to an internal memory and transfer the data to the main computer on request. This has the major drawback that a full-frame, 1.5 s long recording with about 8 GBs of data needs roughly 10 min for data transfer. Therefore, it is impractical to record larger fractions of data of a single subject multiple times, e.g. different phonations, when time is a relevant factor. OpenHSV is potentially able to be extended to support various equipment, for example live streaming of high-speed footage as integrated into the next generation of clinical high-speed videoendoscopy systems^26^. However, as OpenHSV is designed as research tool, OpenHSV has its strength in flexibility and customization.

We found that our preliminary clinical study shows that both, audio and video data can be recorded and successfully analyzed using OpenHSV, having a good agreement between audio and video data (Fig. 8c). As we analyzed 28 healthy individuals, we believe that our data represents general validity, as we show that computed quantitative parameters for audio and video data are of similar magnitude as expected for healthy individuals^36,49,50,54^. However, it remains to be investigated how OpenHSV performs on subjects showing pathologies. As we show that OpenHSV provides a better image quality compared to previous systems (Fig. 8b), we are certain that also organic pathologies and inflammations are at least on par.

As we and others have shown the promise of HSV in analyzing voice pathologies^1,3,5,11,42,43^, we are confident that OpenHSV is another major step forward to disseminate HSV further into research and eventually towards broad clinical application.

## Conclusions

HSV is an important tool to study voice physiology. We contribute OpenHSV, an open system with video and audio acquisition accompanied with data analysis. These unique properties of OpenHSV will enable researchers to conduct HSV studies with latest equipment and image processing technique. Due to the modular nature of OpenHSV, we expect that researchers expand OpenHSV to their individual needs.

## Supplementary Information

Supplementary Information.

Supplementary Video 1.

Supplementary Video 2.

Supplementary Figures.

## Data Availability

The OpenHSV software is available at https://github.com/anki-xyz/openhsv. All further information, including documentation is available on the Github repository. The datasets used and analyzed during the current study are available from the corresponding author upon request.

## Code availability

The OpenHSV code to conduct high-speed videoendoscopy examinations, to analyze the acquired data and to store subject data is available open source at https://github.com/anki-xyz/openhsv. We further provide an in-depth documentation of the code at https://openhsv.readthedocs.io/en/latest/. In the documentation, we explain how to setup the OpenHSV system and perform first recordings. Different cameras can be used in general, when the camera interface is adapted accordingly. To operate OpenHSV for debugging purposes, we added a dummy camera interface that loops through an example video. All algorithms, such as parameter computation, midline prediction and audio analysis can be tested using the example files provided with OpenHSV.
