# Supplementary material for: OpenHSV: an open platform for laryngeal high-speed videoendoscopy
Source: Sci Rep. 2021 Jul 2;11:13760. doi: 10.1038/s41598-021-93149-0 (PMC8253769; doi:10.1038/s41598-021-93149-0)
Supplement: Supplementary file 4 — Supplementary Figures. [file 41598_2021_93149_MOESM4_ESM.pdf]

OpenHSV: An open platform for laryngeal high-speed videoendoscopy

Andreas M Kist, Stephan Dürr, Anne Schützenberger, Michael Döllinger

Supplementary Information

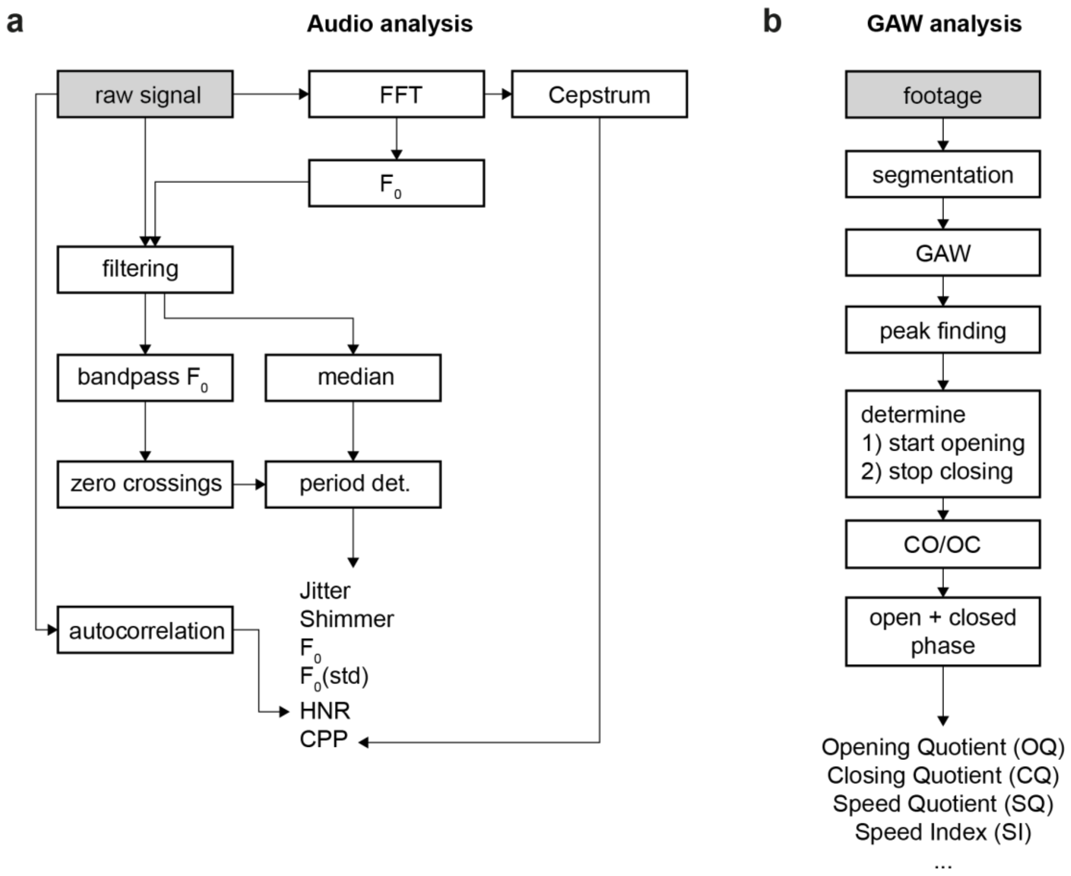

Supplementary Figure 1. Data analysis pipeline for parameter computation.

a) Audio pipeline. b) Glottal area waveform (GAW) pipeline. Source signals are indicated with a gray background in both panels.

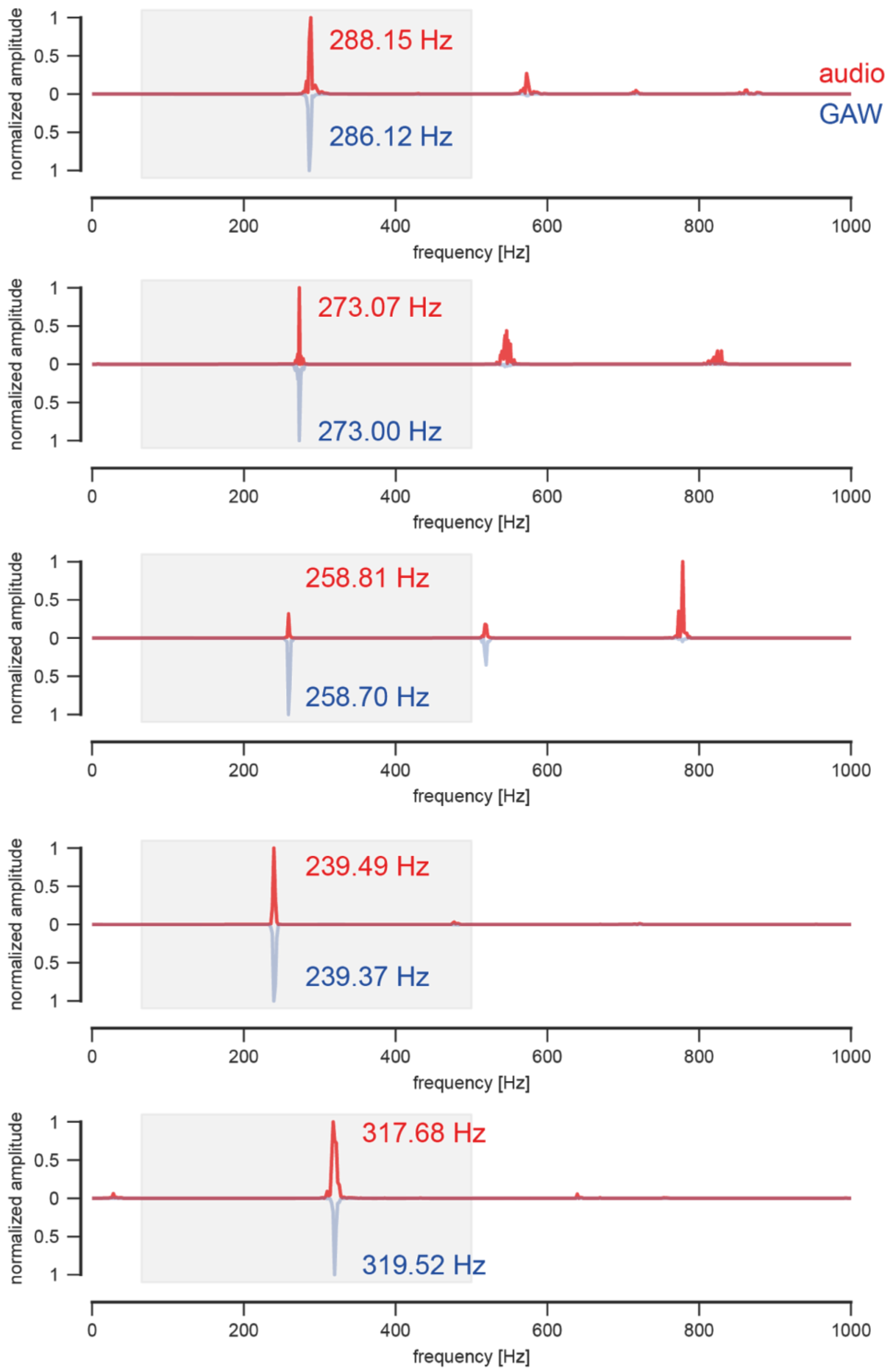

Supplementary Figure 2. Example audio (red) and GAW (blue) power spectra. The gray area indicates boundary conditions for detecting the fundamental frequency (50 to 500 Hz). Spectra were normalized to dominant frequency.

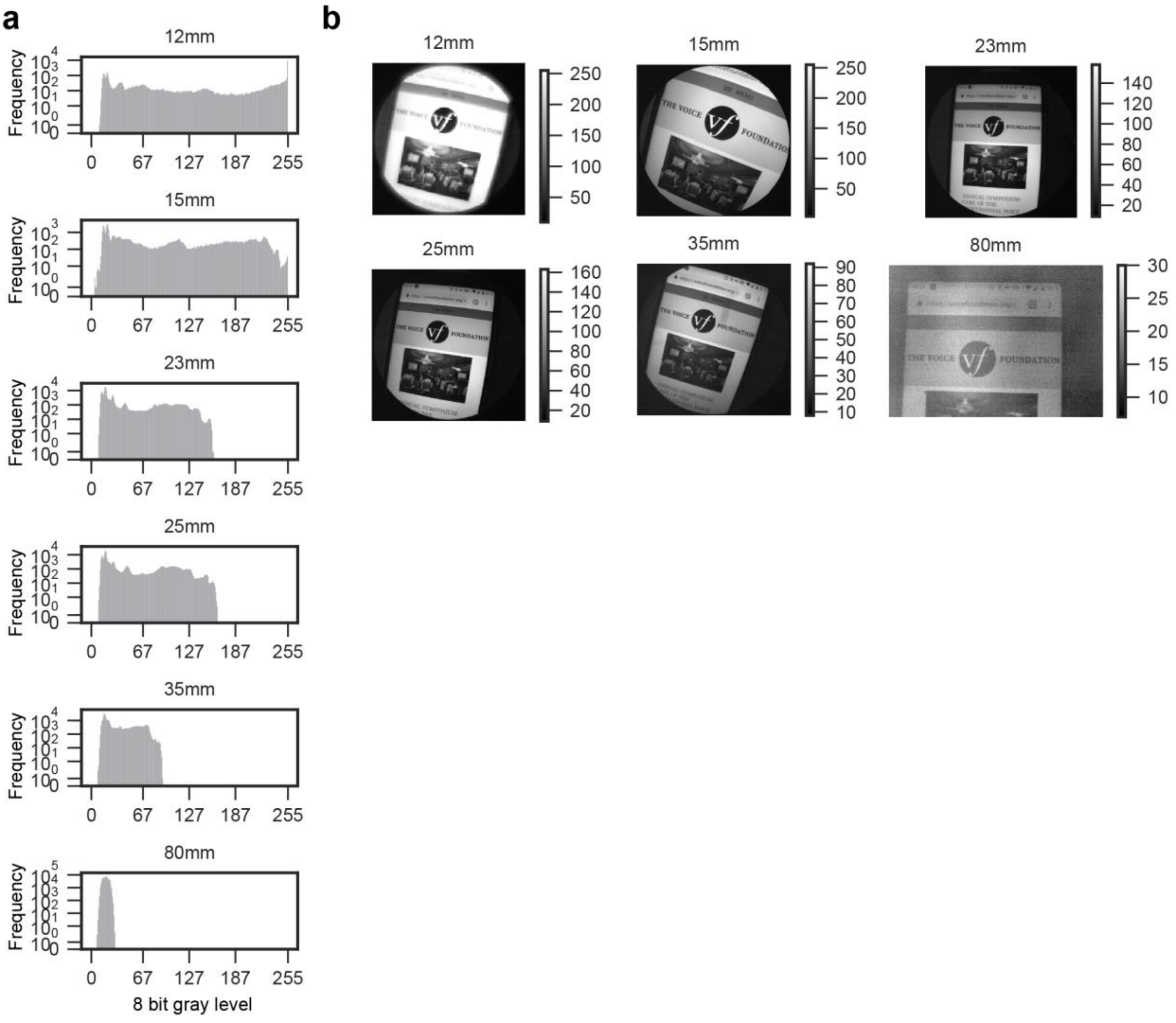

Supplementary Figure 3. Dynamic range of the OpenHSV system. a) Intensity distribution of the luminance channel of the RGB images shown in Figure 4c. **b)** Luminance channel of the RGB images shown in Figure 4c, scaled to maximum contrast and cropped to chip coverage.

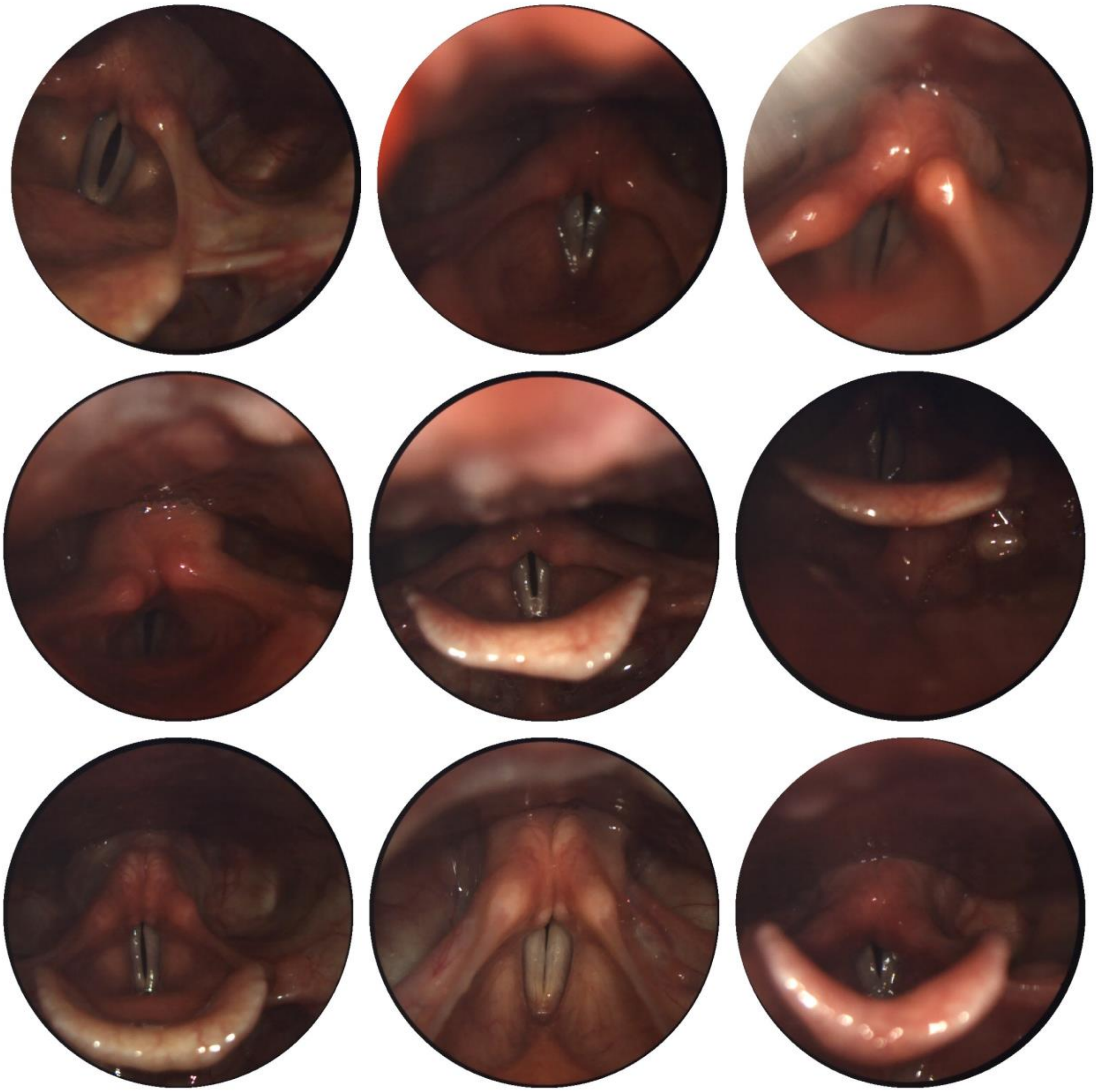

Supplementary Figure 4. Example images of OpenHSV recordings. Background was removed afterwards.
